# The Method of Spread of Gas Gangrene into Living Muscle

**Published:** 1917-12

**Authors:** 


### *The Method of Spread of Gas Gangrene into Living Muscle.*

By Capt. J. W. Mc Nee, R. A. M. C., and J. Shaw Dunn, R. A. M. C. Abs. from the *British Medical Journal*, June 2, 1917.

The authors have never seen gas gangrene commence where injury to muscle could be excluded. Even in cases of metastatic gas gangrene, local muscular damage seems to be the causing agent. No case of gas gangrene beginning in, and remaining localized to, subcutaneous tissue has been noted.

The rapidity of the spread of gas gangrene appears to the authors its most astonishing characteristic. They have found the condition established within three and a half hours after a wound, and they have known of a fatal issue within twelve hours after a thigh wound. They note especially that rapid fatality often results from small wounds with a comparatively small gangrenous bulk of muscle, and they conclude that " the material elaborated by the bacilli, whether it be a true toxin or not, is at any rate a powerful systemic poison ".

After stating that massive gangrene frequently develops in the distal segment of a limb from which the main blood supply has been cut off by the wound, the authors remark that the invasion of living muscle with an intact blood supply is a much more difficult problem, and one which they mean especially to consider in the present paper.

The authors summarize the outstanding facts derived from their own observations and those of others, noting that gas gangrene tends to spread longitudinally from end to end of a single muscle while neighboring muscles remain intact, also that the anaerobic organisms are often to be found in perfectly healthy tissues at a considerable distance from the seat of infection, and when so situated they appear to produce no serious results. Although many organisms are frequently to be found in the flora of gas gangrene, B. aerogenes capsulatus appears to the authors to be the commonest, most abundant, and the most naturally fitted to produce the phenomena observed, mainly because of its powerful fermentative action on sugars and the resulting evolution of gas.

The authors give the results of careful histological examination, especially of a few excellent specimens of long muscle freshly removed from amputated limbs and fixed by varied and careful processes. They note that the spreading margin is sometimes sharp, but in other instances irregular, because the infection spreads more rapidly in some fibers than in others. Although the muscle fibers at the margin are paler than usual, the change is little more than would be due to the total absence of blood. The outer edge of the advance is marked by abrupt loss of contractility and by the firmness of the infected region. This firmness continues back into the definitely gangrenous parts.

Figures 8, 9, and 10 are microscopic drawings of serial transverse sections cut from the entire length of the advancing edge of infection.

Figure 8 represents perfectly healthy muscle just beyond the margin of advance. The cracks in the fibers are artefacts produced in the cutting ot the paraffin sections.

Figure 9 is of a section cut as nearly as possible to the outer limits of the advancing margin as seen by the naked eye. Some of the fibers are normal, while others indicate a marked change. In the affected fibers the dots representing individual fibrils are lost, and the entire surface has stained a uniform eosin tint. The fibers so changed are if anything somewhat swollen, but in spite of this they are separated off from the interstitial connective tissue, leaving what appears to be a clear space. Even where the separation is only partial, the fibers still show the uniform eosin tint. The sarcolemma nuclei still stain brightly. The contents of the spaces is discussed later. Obviously such fibers are cut off from their blood supply.

Figure 10 is of a section 2 1/2 mm. behind the preceding. All the fibers have here undergone the degenerative process. Special attention is called to the regularity of the interstitial net-work. The nuclei of the sarcolemma are, at this stage, stained as in the normal fibers.

Similar changes are to be noted when the fibers are examined longitudinally, the point of demarcation being clearly indicated. The technique of these observations is more difficult.

The authors conclude that the cause of the change from the healthy to the degenerated condition of the fibers is to be sought in the contents of the spaces occurring between the affected fibers and the interstitial tissues. They have therefore sought by various means to determine the nature of this contents. It was tempting to suppose them filled with gas, but all their observations appear to contradict this assumption. They attempted to detect the presence of gas by cutting thick frozen sections of fresh unfixed tissues, and looking for evidence of the gas under the microscope, but none could be observed. When, however, tissues were fixed in corrosive sublimate, and more especially in material fixed by boiling before being embedded in paraffin, evidences of amorphous deposit in the spaces were found, which seemed to point to the contents being a fluid. In tissues fixed in formalin, as in the case of the drawings, the spaces appeared quite empty. The authors therefore conclude that the space is invaded by a toxic fluid similar to the edema that usually accompanies gaseous gangrene. This fluid not only may cause the swelling, but it may also destroy the muscle fibers. When these are once dead the anaerobic bacilli live on them practically as saprophytes, breaking down the sugars and producing abundant gas. Without the presence of this fluid, therefore, the organism is itself powerless to affect healthy tissue. The writers think that this theory fits in with all the phenomena observed, and explains the advance of the infection along the fibers of a single muscle only. They do not, however, venture as yet to express an opinion as to the nature of the toxic fluid, which they believe is formed in the gangrenous part behind the line of advance. It may be a true bacterial toxin or a product of muscle destruction.

Figure 11 shows what the authors believe to be the habits of the organism at the margin of advance. They are to be found never within the muscle fibres at this point, but always confined to the recticulum. When, however, the muscles are being broken down, the bacilli enter them in large numbers.

Figure 13 represents a stage in which the fibres have been completely broken down and all muscle semblance lost. The authors note that gas is abundant in the spaces between muscle fragments, and that the bacilli are less frequently found in this stage, and then merely as ghost forms. They believe this condition to be inimical to thcir presence.

Figure 12 shows abundant phagocytosis going on in a stump where recrudescence was checked after the infection had advanced only a few inches. The authors, however, find few or no leucocytes in muscles, at the margin of rapidly spreading gas gangrene. They believe this absence is due to the rapidity of the advance which does not sufficiently allow for leucocytic reaction to set in. Nevertheless polymorphonuclear leucocytes are abundant and active when the spread of gas gangrene is being arrested in the muscle, as in Figure 12.

Experiments on large rabbits confirm the authors in the beliefs set forth above. The best means of reproducing the disease as observed in man, was to inject about 1 cc. of the fluid expressed from a human muscle in a fairly advanced stage of gas gangrene. Besides abundant bacilli, this fluid contained all the products of tissue disintegration.

When, however, this fluid was freed of its organisms in a Berkefeld V filter, it produced in the animals only a marked local nécrosis without any marked spreading edema or muscle separation.

A rapidly spreading fatal case of gas gangrene was produced by a pure culture of B. perfringens (aerogenes capsulatus) isolated from a fulminating human case. A considerable hematoma at the site of the inoculation may have provided the necessary medium of dead tissue for the start of the infection. Tissues examined microscopically from this animal showed practically all of the conditions indicated in plates 9 and 10. In other cases of inoculation with pure cultures there was produced only a local gangrene, completely walled off by a quickly formed zone of granulation.

The authors conclude that the rapid spread of gas gangrene is due to the peculiarities of muscle structure, the sheaths of muscle " being so easily detachable as to form potential spaces into which toxic material can readily pass, causing necrosis of the fibers."

